# Catalytic Hydrogen Evolution of NaBH_4_ Hydrolysis by Cobalt Nanoparticles Supported on Bagasse-Derived Porous Carbon

**DOI:** 10.3390/nano11123259

**Published:** 2021-11-30

**Authors:** Yiting Bu, Jiaxi Liu, Hailiang Chu, Sheng Wei, Qingqing Yin, Li Kang, Xiaoshuang Luo, Lixian Sun, Fen Xu, Pengru Huang, Federico Rosei, Aleskey A. Pimerzin, Hans Juergen Seifert, Yong Du, Jianchuan Wang

**Affiliations:** 1 Guangxi Key Laboratory of Information Materials and Guangxi Collaborative Innovation Center of Structure and Property for New Energy and Materials, School of Material Science & Engineering, Guilin University of Electronic Technology, Guilin 541004, China; ytb1172701255@163.com (Y.B.); jxliu2019@126.com (J.L.); chuhailiang@guet.edu.cn (H.C.); ws1801101003@163.com (S.W.); yqq15870030656@163.com (Q.Y.); kangli000hello@163.com (L.K.); shirley_lxs@126.com (X.L.); pengruhuang@guet.edu.cn (P.H.); 2 School of Mechanical & Electrical Engineering, Guilin University of Electronic Technology, Guilin 541004, China; 3 Department of Materials Science and Engineering, National University of Singapore, Singapore 117575, Singapore; 4 Centre for Energy, Materials and Telecommunications, Institut National de la Recherche Scientifique, 1650 Boulevard Lionel-Boulet Varennes, Québec, QC J3X 1S2, Canada; rosei@emt.inrs.ca; 5 Chemical Department, Samara State Technical University, 443100 Samara, Russia; al.pimerzin@gmail.com; 6 Karlsruhe Institute of Technology, Institute for Applied Materials-Applied Materials Physics, Hermann-von-Helmholtz-Platz 1, 76344 Eggenstein-Leopoldshafen, Germany; hans.Seifert@kit.edu; 7 State Key Laboratory of Powder Metallurgy, Central South University, Changsha 410083, China; yong-du@csu.edu.cn (Y.D.); jcw728@126.com (J.W.)

**Keywords:** sodium borohydride, hydrolysis, porous carbon, Co nanoparticles, durability

## Abstract

As a promising hydrogen storage material, sodium borohydride (NaBH_4_) exhibits superior stability in alkaline solutions and delivers 10.8 wt.% theoretical hydrogen storage capacity. Nevertheless, its hydrolysis reaction at room temperature must be activated and accelerated by adding an effective catalyst. In this study, we synthesize Co nanoparticles supported on bagasse-derived porous carbon (Co@xPC) for catalytic hydrolytic dehydrogenation of NaBH_4_. According to the experimental results, Co nanoparticles with uniform particle size and high dispersion are successfully supported on porous carbon to achieve a Co@150PC catalyst. It exhibits particularly high activity of hydrogen generation with the optimal hydrogen production rate of 11086.4 mL_H2_∙min^−1^∙g_Co_^−1^ and low activation energy (*E_a_*) of 31.25 kJ mol^−1^. The calculation results based on density functional theory (DFT) indicate that the Co@xPC structure is conducive to the dissociation of [BH_4_]^−^, which effectively enhances the hydrolysis efficiency of NaBH_4_. Moreover, Co@150PC presents an excellent durability, retaining 72.0% of the initial catalyst activity after 15 cycling tests. Moreover, we also explored the degradation mechanism of catalyst performance.

## 1. Introduction

Two of the seventeen Sustainable Development Goals (SDGs), namely 7 (affordable and clean energy) and 13 (climate action), dictate the urgency of transitioning from highly polluting and non-sustainable fossil fuels to renewable energy sources [1,2,3]. Hydrogen is considered a very promising green new energy carrier because of its high-energy and zero-emission applications [4,5,6,7]. However, the physical properties of hydrogen make it have high energy consumption and high risk for application on-board. Therefore, we need to find alternative ways to store and transport H_2_. Compared with high-pressure gas storage and cryogenic liquid storage, solid state hydrogen storage materials [8] such as MgH_2_, NaAlH_4_, NaBH_4_, etc., have attracted more and more attention and research due to high hydrogen capacity, safe operation and relative abundance.

Among many hydrogen storage materials, NaBH_4_ is considered to be a promising one because of its theoretical hydrogen storage capacity of 10.8 wt.% and good stability in alkaline solutions [9]. Nevertheless, the hydrogen production rate is very low and unsatisfactory without a proper catalyst at room temperature [10]. Therefore, it is necessary to activate and accelerate the NaBH_4_ hydrolysis reaction at room temperature through the use of high-efficiency catalysts. Guella et al. [11] followed the reaction of NaBH_4_ in the presence of catalysts and its perdeuterated analogue NaBD_4_ in H_2_O, D_2_O and H_2_O/D_2_O mixtures. The results revealed that NaBH_4_ can react with water to generate hydrogen (Equation (1)), of which four equivalents of H come from BH_4_^−^, and four equivalents of D from the decomposition of D_2_O. The reaction formula is summarized as follows:

(1) $${\mathrm{BH}}_{4}^{-}+4D_{2}O\to B{\left(\mathrm{OD}\right)}_{4}^{-}+4\mathrm{HD}$$

In recent years, relatively inexpensive transition metals [12] (such as Co [13], Ni [14], Cu [15] and Fe [16]) have been observed to exhibit excellent performances in catalytic sodium borohydride hydrolysis, especially metal nanoparticles prepared with transition metals as catalysts. In particular, Co-based catalysts are considered to be particularly attractive in the hydrolysis of NaBH_4_ for hydrogen production due to their high activity and relatively low cost [17,18,19]. Metal catalysts are usually prepared by chemical reduction using NaBH_4_ and ammonia borane. However, metal nanoparticles are prone to agglomeration during the reduction and catalytic process, resulting in reduced catalyst activity and poor cycle stability [20]. In order to prevent agglomeration, the most used method is to choose suitable support materials, including SiO_2_ [21], γ-Al_2_O_3_ [22], hydroxyapatite [23], carbon materials [24,25], etc. In particular, the addition of appropriate promoters could be used to enhance the dispersibility of Co nanoparticles and increase the surficial active sites [18]. Therefore, using the reduction method, the surface of supporting materials could be loaded with reduced Co nanoparticles, so that the agglomeration of the composite catalyst is inhibited and its specific surface area increased. Compared with unsupported metal catalysts, catalysts composed of support materials and metal particles have a larger specific surface area [26], which increases the contact area between metal particles and NaBH_4_ aqueous solution. In addition, supporting materials increase the stability of metal particles, greatly increasing the durability [27,28,29]. Among the supporting materials mentioned above, carbon materials such as porous carbon (PC) are the most attractive support due to their chemical inertness and large surface area [24]. At present, the reported porous carbon materials [30] loaded with Co nanoparticles mainly include carbon nanotubes, graphene, activated carbon, organic drugs [31] and polymer materials [13]. Among them, carbon nanotubes, graphene, activated carbon and polymer materials are relatively expensive. Meanwhile, most organic drugs are also not conducive to practical promotion because they contain a certain level of toxicity. Therefore, PC prepared from biomass is an excellent candidate as a support because it not only has a wide range of raw materials (especially biomass such as bagasse) but is also simple to prepare.

In our previous work [31], we synthesized nitrogen-doped mesoporous graphitic carbon-coated cobalt nanoparticles (Co@NMGC) with a core–shell structure by carbonizing carbon derived from ethylenediaminetetraacetic acid (EDTA). However, the specific surface area of Co@NMGC is only 124.55 m^2^·g^−1^, and the cobalt nanoparticles coated with graphite carbon cannot effectively contact and react with NaBH_4_, resulting in an optimal hydrogen production rate of Co@NMGC of only 3575 mL min^−1^ g^−1^. Based on these considerations, using bagasse as raw material for PC, we designed and prepared a kind of PC to load Co nanoparticles (Co@xPC) for NaBH_4_ hydrolysis. The structural characteristics and catalytic performance of several PC-supported Co nanoparticle catalysts were studied in detail. After optimization, a Co-based catalyst with high efficiency, excellent activity and high durability was achieved. The Co@xPC structure was beneficial to improve the hydrolysis efficiency of NaBH_4_, confirmed by the theoretical calculation based on density functional theory (DFT).

## 2. Materials and Methods

### 2.1. Materials

Bagasse was collected from fruit shops on East West Street of Guangxi, China, which have abundant sugarcane. We purchased sodium borohydride (NaBH_4_, AR), ZnCl_2_ (AR), cobalt(II) chloride hexahydrate (CoCl_2_·6H_2_O, AR) and Mg(NO_3_)_2_·6H_2_O (AR) from Alfa Aesar Co., Ltd. (Tianjin, China). Hydrochloric acid (HCl) was from Xilong Chemical Co., Ltd. (Shantou, China). Ultrapure water, obtained from a Millipore System (Millipore Q, Burlington, MA, USA), was used throughout the experiments.

### 2.2. Synthesis of Co@xPC Catalyst

Bagasse was freeze-dried with a freezer, then crushed with a pulverizer. The crushed bagasse powder and activator were completely ground in a mortar according to the mass ratio (1:2). The activator was a mixture of Mg(NO_3_)_2_·6H_2_O and ZnCl_2_ with a mass ratio of 1:1. The well-ground mixture was moved to a crucible and kept at 800 °C for 2 h at a heating rate of 3 °C min^−1^ under N_2_ flow. The resulting black sample was soaked in 3 M HCl at 80 °C for 24 h to remove inorganic salts. Then, the black sample was washed with ultrapure water to neutrality and dried at 100 °C for 24 h, and the resulting sample was named PC. In the preparation process, appropriate CoCl_2_·6H_2_O was dissolved in 20 mL ultrapure water, and different amounts of PC (50, 100, 150 and 200 mg) were added at the same time. The above solution was treated under ultrasonic conditions for 1 h, and then 20 mL 3 wt.% NaBH_4_ solution was slowly added under constant stirring. The black solid was separated with vacuum suction filtration and washed with ethanol and ultrapure water several times, respectively. The sample was dried in a vacuum oven at 60 °C. According to the different amounts of added PC, the corresponding catalysts were marked as Co@50PC, Co@100PC, Co@150PC and Co@200PC, respectively. At the same time, pure Co particle catalysts without PC were also prepared in the same conditions for comparison.

### 2.3. Catalyst Characterization

The morphology, surface structure and element distribution of the catalysts were analyzed through scanning electron microscopy (SEM, JSM-6360LV, JEOL Ltd., Tokyo, Japan) combined with energy dispersive spectroscopy (EDS) at 20kV. The surface interactions and electronic states between the elements of the catalyst were obtained by using X-ray photoelectron spectroscopy (XPS, Thermo Electron ESCALAB 250, Shanghai, China). The nitrogen adsorption–desorption isotherms of the Co@xPC catalysts were tested at 77 K using a gas adsorption analyzer (Autosorb iQ2, Quantachrome sorptometer, Osaka, Japan). The specific surface areas and pore size distribution of the Co@xPC catalysts were obtained by the Brunauer–Emmett–Teller (BET) method and the Barrett–Joyner–Halenda (BJH) method, respectively. The crystallographic structure and chemical composition of the catalysts were obtained by X-ray diffraction (XRD, 1820, Philips, Amsterdam, the Netherlands), inductively coupled plasma atomic emission spectroscopy (ICP-AES, Optima 8000, PerkinElmer, Chiba, Japan), laser confocal Raman spectroscopy (LabRAM HR Evolution, Horiba JY, Edison, NJ, USA) and Fourier transform infrared spectroscopy (FT-IR, Nicolet 6700, Shanghai, China).

### 2.4. Measurement Method of Hydrogen Production

The catalytic activity of Co@xPC was evaluated by measuring the hydrogen production rate of Co@xPC in alkaline NaBH_4_ hydrolysis. In the self-made reactor, the classic water displacement method was used to measure the hydrogen production rate of NaBH_4_, as in our previous work [31]. The volume at constant time intervals was determined by using a balance to record the weight of the replaced water [31,32], and the volume of hydrogen produced was measured in an equivalent way. Usually, a magnetic rotor and 0.1 g Co@xPC catalyst were placed at the bottom of a wide-mouthed bottle. At 25 °C, 10 mL mixed solution containing 1.5 wt.% NaBH_4_ and 5.0 wt.% NaOH was quickly injected into the wide-mouthed bottle, and a constant temperature water bath was used to maintain the test system at a constant temperature. The tests were carried out at 15, 25, 35, 45 and 55 °C, and other experimental conditions remain unchanged to obtain E_a_. In the stability tests, the reaction was repeated 15 times, and the reacted catalyst was named Co@xPC-15th. Similarly, the reaction of pure Co particle catalyst was repeated 5 times, and the reacted catalyst was named Co-5th. In the stability test, after the hydrolysis was over, the supernatant was poured from the wide-mouthed bottle and then 10 mL NaBH_4_ alkali solution was added to start the next test. Since the catalyst was magnetic, a magnet was used to recover the catalyst after the hydrolysis reaction was completed. During the experiment, the hydrogen generation rate for all catalysts was based on the amount of Co.

### 2.5. DFT Calculations

In this work, all calculations are performed with DFT [33] using the projected augmented wave method, which was implemented in the Vienna Ab-initio Simulation Package [34,35,36]. The generalized gradient approximation of Perdew–Burke–Ernzerhof was used for the exchange–correlation interaction [37,38]. The wave functions are expressed in the plane wave basis set with an energy cutoff of 450 eV. A vacuum region of 15 Å was set to eliminate undesirable interactions between the periodic sheets of the graphene patches. The Brillouin zone was sampled by a Monkhorst–Pack special k-point mesh of 5 × 5 × 1 for geometry optimizations and energy calculations. All atoms were allowed to relax until the final energy and the forces on each atom converged to 10^−4^ eV and 0.02 eV/Å, respectively. The quantitative charge changes of the Co_4_ and graphene patch were described using a grid-based Bader charge transfer analysis method [39]. The adsorption energy was calculated as follows:

(2) $$E_{ads}=E_{total}-E_{graphene}-E_{Co_{4}}$$

where *E*_*Co*4_ is the calculated energy of the Co_4_ cluster, *E_graphene_* is the energy of the graphene surface and *E_total_* is the total energy of the absorption system that contains both the cluster and the graphene patch.

During the hydrolysis process, the remaining one or more hydrogen atoms from the previous step are accepted when far away from the central unit cell of the surface. As the total number of atoms in the system must be conserved in order to generate a relative energy diagram, thus the infinite distance approach (IDA) was considered in the calculation. IDA energy is equal to the number of the adsorbed hydrogen atom(s), and it can be defined as follows [40]:

(3) $$E_{IDA}=E_{H/slab}-E_{slab}$$

where *E_H/slab_* is the total energy of the hydrogen adsorbed slab and *E_slab_* is the total energy of the pure slab.

## 3. Results and Discussion

### 3.1. Characterization of the as-Prepared Catalysts

**Figure 1:**
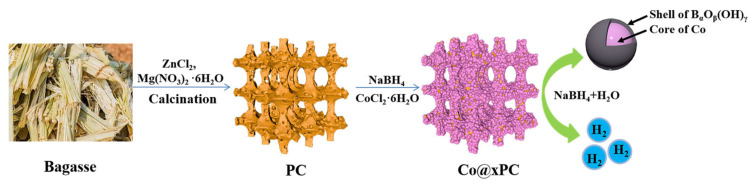
Schematic diagram of the preparation and catalytic process of Co@xPC.

First, the PC was prepared by carbonizing the powder mixture of bagasse, zinc chloride and magnesium sulfate at 800 °C for 2 h under N_2_ flow with a heating rate of 3 °C min^−1^. Then, the PC was dispersed in the aqueous solution by ultrasound. Under stirring conditions, cobalt chloride hexahydrate was added to the suspension so that Co^2+^ was well dispersed around the PC. After that, the NaBH_4_ aqueous solution was slowly dropped into the system to reduce Co^2+^ to Co on the PC surface (Figure 1). Finically, the catalysts of Co@50PC, Co@100PC, Co@150PC and Co@200PC were obtained according to the different amounts of PC (50, 100, 150 and 200 mg).

**Figure 2:**
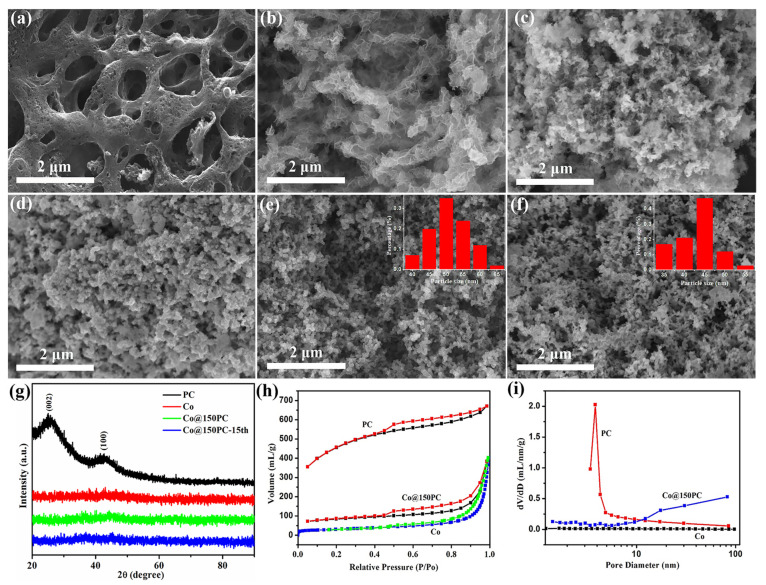
SEM images of PC (**a**), Co (**b**), Co@50PC (**c**), Co@100PC (**d**), Co@150PC (**e**) and Co@200PC (**f**). XRD patterns of the as-prepared catalysts (**g**). N_2_ adsorption–desorption isotherms (**h**) and corresponding BJH pore-size distribution plots (**i**) of PC, Co and Co@150PC. The insets in (**e**,**f**) are the size distribution of metal Co nanoparticles.

In Figure 2a, the PC shows a clear porous structure on its surface and on the scaffold. Co nanoparticles are flaky and aggregate into branches (Figure 2b). After supporting Co nanoparticles (Figure 2c–f), with the increase in the addition of PC, the dispersibility of Co nanoparticles in Co@xPC was significantly enhanced and gradually changed from the mixed state of flakes and particles to Co nanoparticles with uniform size. Compared with the agglomerated Co in Figure 2b, the dispersion of Co in Co@xPC has been significantly improved. Particularly in Co@150PC and Co@200PC, Co nanoparticles were evenly dispersed on the surface of PC (Figure S1) without obvious agglomeration (Figure 2e–f). The average particle size of Co nanoparticles in Co@150PC and Co@200PC was determined to be 50 and 45 nm, respectively, much smaller than that in Co, Co@50PC and Co@100PC samples. Furthermore, compared with Co nanoparticles in Figure 2b, the presence of PC support could effectively prevent the agglomeration of catalysts in the preparation and catalytic reaction process, resulting in a smaller size and more even dispersion of Co particles. Through careful examination, the Co nanoparticles of Co@150PC (51.82 wt.%) were found to be more uniform than Co@200PC (44.65 wt.%) and the relative content of Co was higher in Co@150PC, confirmed by ICP analysis. The XRD patterns of PC, Co, Co@50PC, Co@100PC, Co@150PC, Co@200PC and Co@150PC-15th are shown in Figure 2g and Figure S2. For the PC, two broad and weak peaks observed at approximately 24° and 42° are attributed to the diffraction of the (002) and (100) planes of graphite [41], respectively. For the neat Co nanoparticles and Co@xPC samples, no observable peaks for the metallic cobalt are found, ascribed to the amorphous state of metallic cobalt [42]. A similar phenomenon was also observed in the XRD patterns of phosphorus-modified spirulina microalgae strains supporting a Co-B catalyst [19]. As described before, Co@150PC exhibits more uniform Co nanoparticles (Figure S3) and relatively high Co content among the as-prepared catalysts. Therefore, only Co@150PC is characterized in the following parts. As shown in Figure 2h, a typical type IV isotherm is observed for the PC, Co and Co@150PC catalysts. The specific surface area (Table 1) of PC was determined to be 1527.5 m^2^·g^−1^ with a pore-size distribution in a narrow 3–4 nm range (Figure 2i). The specific surface areas of the Co and Co@150PC catalysts were 87.1 and 274.1 m^2^·g^−1^, respectively. Therefore, the addition of PC effectively increases the specific surface area of Co@150PC.

**Figure 3:**
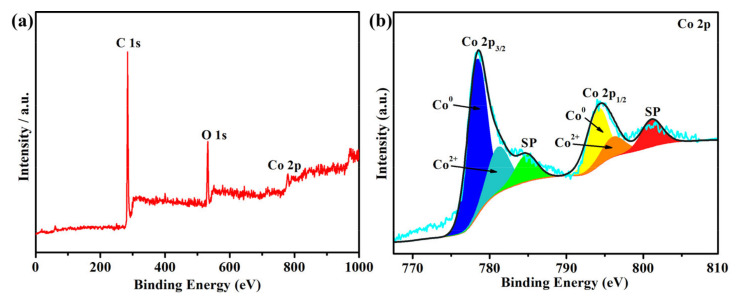
XPS spectra of Co@150PC: (**a**) survey spectrum, (**b**) Co 2p spectrum.

From Figure 3a, the C 1s and Co 2p photoionization signals are observed in the XPS survey spectrum, which confirmed the presence of boron, carbon and cobalt species in the Co@150PC. The binding energy of the C 1s spectrum in Figure S4 can be divided into three fitting peaks at 284.8 eV, 286.2 eV and 288.9 eV, which are, respectively, related to C-C, C-O and C=O [43,44,45]. In Figure 3b, the Co 2p spectrum shows the six fitting peaks. The peaks of 778.8 eV (Co 2p_3/2_) and 793.6 eV (Co 2p_1/2_) correspond to the reduced metallic Co in the Co@150PC. Two peaks were observed at 780.8 eV (Co 2p_3/2_) and 796.4 eV (Co 2p_1/2_), which corresponded to the spin-orbit peaks of Co 2p_3/2_ and Co 2p_1/2_ of CoO [31]. This indicated the presence of CoO in the Co@150PC catalyst. Moreover, satellite peaks corresponding to CoO were also observed at 786.2 eV and 802.8 eV. The quantitative analysis of the Co 2p XPS spectrum indicated that the Co^0^:Co^2+^ atomic ratio was 6.21:1.00, showing that most of the cobalt in Co@150PC exists in a reduced state. In summary, Co nanoparticles in Co@150PC mainly existed as metallic cobalt and also contained a small amount of CoO, which possibly resulted from cobalt nanoparticles easily combining with atmospheric oxygen in the preparation and storage of the catalyst [17].

### 3.2. Catalytic Activity Tests of Co@xPC for Hydrolysis of NaBH_4_

**Figure 4:**
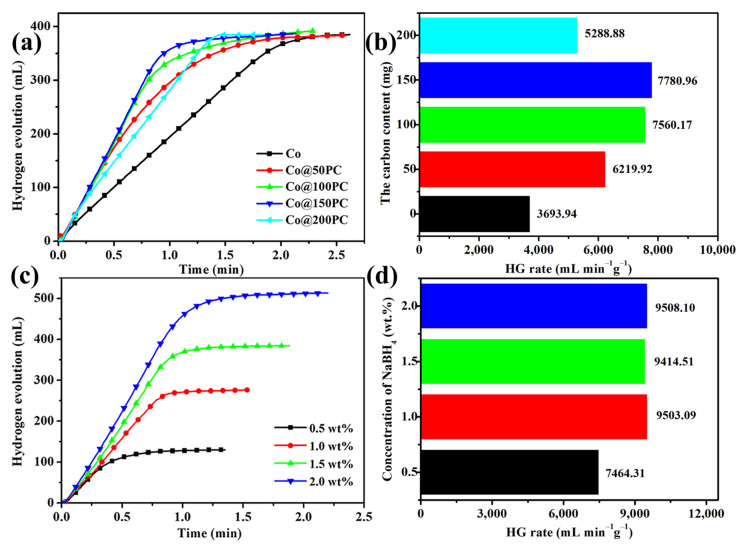
Hydrogen production rate of the as-prepared samples with (**a**,**b**) amount of PC added in Co, (**c**,**d**) NaBH_4_ concentration.

The NaBH_4_ hydrolysis performance catalyzed by Co and Co@xPC was tested under the same conditions to explore the influence of the addition of PC on the catalytic performance of Co (Figure 4a–b). The hydrogen production rate was only 3693.94 mL_H2_∙min^−1^∙g_Co_^−1^ when no PC was added. This low value was probably obtained due to the severe agglomeration of Co particles without support materials (Figure 2b). This caused a decrease in the number of active sites on the Co surface, which in turn affected the catalytic performance. Correspondingly, when increasing the amount of PC, the hydrogen production rate of Co@xPC first increased rapidly because the presence of PC can effectively inhibit the agglomeration of Co (Figure 2c–f). Then, the hydrogen production rate suddenly decreased after reaching a maximum value with Co@150PC. This may be because the amount of PC added was too high, and the Co content in Co@200PC was reduced too much, slowing down its catalytic rate of NaBH_4_. Compared with previous work (Table 2), the addition of PC with a large specific surface area obtained from bagasse is more effective than other carbon materials in improving the performance of Co nanoparticles.

The effect of different NaBH_4_ concentrations on the hydrogen production rate catalyzed by Co@150PC catalyst with a Co loading of 51.82 wt.% (characterized by ICP) was studied. In Figure 4c,d, it can be seen that when the concentration of NaBH_4_ was 0.5 wt.%, the reaction rate was significantly slower due to the low concentration of NaBH_4_. However, as the concentration of NaBH_4_ increased, the hydrogen production rate did not increase significantly, which indicated that the concentration of NaBH_4_ had no obvious effect on the hydrolysis catalyzed by Co@150PC. Therefore, a zero-order reaction is ascribed to the hydrolysis of NaBH_4_ to produce hydrogen using a Co@150PC catalyst [57,58] and the rate-determining step should have been the hydrolysis reaction [59].

**Figure 5:**
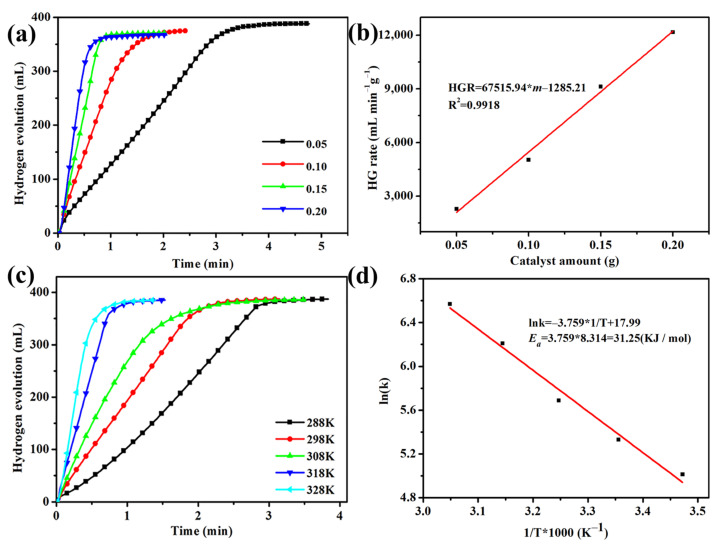
Hydrogen production rate of the as-prepared samples with (**a**,**b**) catalyst amount, (**c**) hydrogen generation kinetics curves employing Co@150PC at different solution temperatures and (**d**) Arrhenius plot.

The hydrogen production rate of NaBH_4_ catalyzed by different amounts of Co@150PC (0.05, 0.10, 0.15 and 0.20 g) was also tested. Figure 5a shows the relationship between the hydrogen production rate and the amount of catalyst added, indicating that the time required to complete the reaction decreases rapidly as the amount of catalyst added increases. Moreover, the rate of the NaBH_4_ hydrolysis reaction shows a good linear fit with respect to the amount of catalyst added (Figure 5b).

To further explore the catalytic activity of the Co@150PC catalyst, Co@150PC catalyst (100 mg) was used to hydrolyze NaBH_4_ at different temperatures from 15 °C to 55 °C. Figure 5c shows that the rate of hydrogen generation increases significantly with the increase in temperature. The activation energy (*E_a_*) of the NaBH_4_ hydrolysis reaction catalyzed by Co@150PC can be obtained from the Arrhenius equation:

(4) $$\ln k=\ln A-(\frac{E_{a}}{RT})$$

According to the linear slope in Figure 5d, *E_a_* for the NaBH_4_ hydrolysis reaction catalyzed by the Co@150PC catalyst was calculated to be 31.25 kJ mol^−1^, lower than those of most reported Co-based catalysts (Table 2).

### 3.3. Catalytic Stability Tests of Co@150PC for Hydrolysis of NaBH_4_

**Figure 6:**
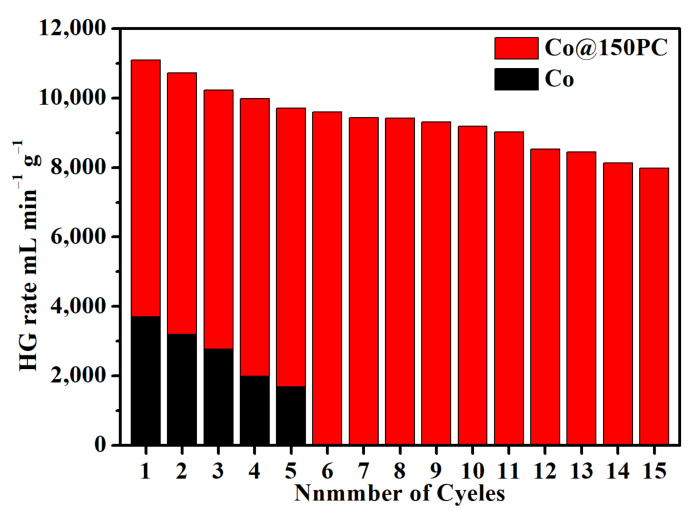
Histogram of hydrogen generation rate and the different cycle times of Co@150PC and Co in the cycle test.

In addition to having excellent catalytic activity, low cost and environmentally friendly properties, the hydrolysis catalyst is also required to have good stability. Therefore, the stability studies of Co@150PC catalyst were conducted. The histogram of the hydrogen generation rate and the different cycle times was drawn to study the changes in the hydrogen generation rate during the stability test (Figure 6). Obviously, the hydrogen generation rate decreased slowly as the number of tests increased. The hydrogen production rate of the Co@150PC catalyst is 7982.54 mL_H2_∙min^−1^∙g_Co_^−1^ at the 15th cycle with a 72.0% hydrogen generation rate of the initial cycle retained. Compared with previous reported values (Table 2), the cycle stability of Co@150PC is significantly improved and the decline in performance is relatively small compared with the Co-based catalysts supported by other carbon materials. To prove that the presence of PC could effectively improve the cycling performance of the catalyst, we performed a repeatability study on the pure Co catalyst under the same conditions (Figure 6). The hydrogen production rate catalyzed by neat Co was 1684.30 mL_H2_∙min^−1^∙g_Co_^−1^ after five hydrolysis cycles, which was only 45.6% of the hydrogen generation rate of the first cycle.

**Figure 7:**
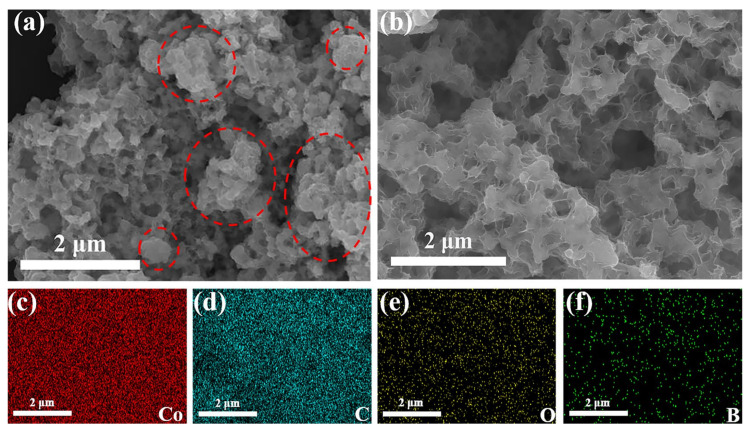
SEM images of Co-5th (**a**), Co@150PC-15th (**b**). Corresponding EDS mapping of Co@150PC-15th (**c**–**f**).

SEM images showed that the structure of Co was agglomerated from flakes into a large number of irregular blocks (Figure 7a). Moreover, compared with Co@150PC-15th (Figure 7b), Co-5th has poor dispersion and uneven size. Therefore, PC effectively inhibits the agglomeration of Co during the hydrolysis reaction process, which reduces the decrease in the number of active sites on the catalyst surface, thus ensuring the high stability of the catalytic performance of Co@150PC.

### 3.4. DFT Calculations of Co@150PC

**Figure 8:**
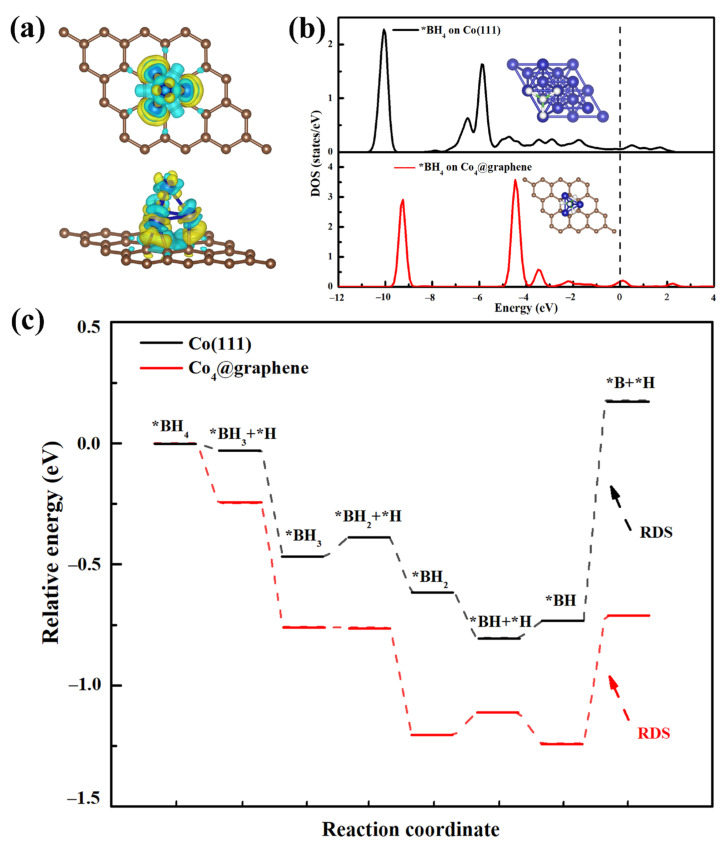
(**a**) Different charge densities of Co_4_@graphene. The isovalue value is 0.005 e/Å^3^, where the cyan and yellow regions indicate a charge depletion and accumulation, respectively. (**b**) Density of states of the absorbed *BH_4_ on Co(111) and Co_4_@graphene. The dashed line indicates the Fermi level. (**c**) Potential energy diagram of Co(111) and Co_4_@graphene.

By constructing a model of Co_4_ clusters on graphene (named Co_4_@graphene) to perform DFT calculations, we can further understand the catalysis of the Co@xPC structure in the NaBH_4_ hydrolysis process. Although the models of Co_4_ clusters and graphene in this experiment are much smaller than the observed Co@xPC nanoparticles, the relative energy of the NaBH_4_ hydrolysis process can be discussed. Therefore, the sequential dissociation of BH_x_ (x = 0→4) molecules on the Co(111) and Co_4_@graphene surface was calculated. First, the structure optimization showed that Co_4_ clusters could be anchored on the graphene surface to maintain a stable structure with an adsorption energy of −3.34 eV. As shown in different charge density maps (Figure 8a), there is a charge accumulation between graphene and the Co_4_ cluster. The graphene gains 0.794 electrons from the Co_4_ cluster, indicating that the redistribution of the electron potential of the Co_4_@graphene structure is weakening the inert B-H bond and activating [BH_4_]^−^. From the density of states (DOS) plots, an increased electron state of *BH_4_ DOS at the Fermi level on Co_4_@graphene compared with the Co(111) surface can be observed (Figure 8b), indicating that the electrons in Co_4_@graphene can efficiently back donate to the unoccupied orbital of [BH_4_]^−^, thus activating the [BH_4_]^−^ molecule. According to Figure 8c, the rate-determining step (RDS) is the BH dissociation step common to both systems in the pathways. Obviously, H abstracting from * BH on the Co(111) and Co_4_@graphene surface is endothermic, which requires 0.911 and 0.527 eV of energies, respectively. Hence, the above results fully suggested that the Co_4_@graphene structure is favorable to the dissociation of [BH_4_]^−^ molecules.

### 3.5. Mechanism Analysis on Performance Decrease of Co@150PC during Cycles

**Figure 9:**
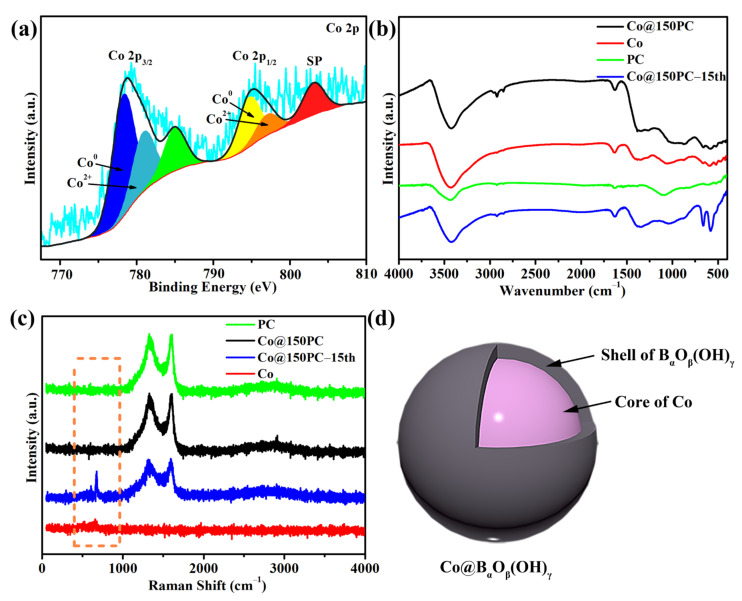
Co 2p XPS spectrum of Co@150PC-15th (**a**). FT-IR spectra (**b**), Raman spectra of the as-prepared catalysts (**c**). Schematic diagram of the structure of Co@B_α_O_β_(OH)_γ_, a core-shell structure with metallic cobalt as the core and (poly)borate as the outer shell (**d**).

To explore the reasons for the decrease in catalyst stability, the used catalysts were characterized after 15 cycles. The dispersed Co particles in Figure 2e have obvious agglomeration after 15 tests. As shown in Figure 7b, the Co nanoparticles on the porous carbon surface became significantly larger, sticking to each other and agglomerating together. By comparing the EDS mappings of Co@150PC (Figure S3) and Co@150PC-15th (Figure 7f), it was found that the B content in Co@150PC-15th increased significantly. ICP-AES analysis further found that the content of B in Co@150PC increased from 0.41 wt.% to 10.9 wt.% after 15 cycles. This indicates that as the number of cycles increased, the content of B continued to increase. At the same time, a Co^0^:Co^2+^ atomic ratio is determined to be 1.90:1.00 according to XPS of Co 2p in Co@150PC-15th (Figure 9a). That is to say, most of the cobalt in Co@150PC-15th exists in a reduced state. In the XRD pattern (Figure S5), compared with Co@150PC, Co@150PC-15th also did not show the characteristic peak of Co-B around 46°. This is probably because of the presence of heat in the reaction during the test and because the surface of Co nanoparticles is covered by a film consisting of strongly adsorbed (poly)borates [46].

To verify the above results, we conducted FT-IR and Raman tests. In the FT-IR spectra (Figure 9b), we can see that at 525–630 cm^−1^, 1349 cm^−1^ and 875 cm^−1^, Co, Co@150PC and Co@150PC-15th all have pulse vibrations, indicating that they all contain borate [60,61]. Among them, the weaker peak intensity of Co and Co@150PC indicates that these untested catalysts have less borate content, and the peak intensity of Co@150PC-15th also proved that the borate content is higher with the increase in cycles of the experiments. Similarly, in the Raman spectra (Figure 9c), Co@150PC-15th had an obvious Raman characteristic peak [60] of B(OH)_4_^−^ at 754 cm^−1^, while Co and Co@150PC were present in negligible quantities.

On this basis we infer that Co nanoparticles in the prepared catalyst first existed in the form of metallic cobalt. As the number of experiments increased, the borate content on the surface of the Co nanoparticles gradually increased, forming a layer of a strongly adsorbed (poly)borate shell (Figure 9d), similarly to previous observations [13,47,62]. The existence of this shell reduces the contact area between Co nanoparticles and NaBH_4_. At the same time, the mutual fusion between the strongly adsorbed (poly)borate shells on the surface of adjacent Co nanoparticles makes the Co nanoparticles stick together, which reduces the dispersion of Co nanoparticles and further affects the catalytic performance of Co@150PC.

Based on the above research, it is found that Co@150PC catalyst can significantly improve the hydrolysis performance of NaBH_4_. It is attributable to the supporting and inhibiting agglomeration effect of PC with a large specific surface area on Co nanoparticles during the synthesis of Co@150PC catalyst, which effectively reduces the particle size of Co particles and distributes them more uniformly to provide more active sites. Similarly, due to the role of PC with a large specific surface area on the loading of Co nanoparticles and the inhibition of agglomeration, the catalyst Co@150PC can effectively retain the good catalytic performance. The calculation results of DFT also proved that Co nanoparticles can be anchored on the surface of PC to maintain a stable structure, and the Co@xPC structure is conducive to the dissociation of [BH_4_]^−^ molecules to realize the rapid water release of hydrogen from NaBH_4_.

## 4. Conclusions

The Co@xPC catalyst was synthesized to catalyze NaBH_4_ hydrolysis for hydrogen evolution. During the synthesis of the Co@xPC catalyst, the PC support can inhibit the agglomeration of Co nanoparticles, which effectively reduced the particle size of the Co nanoparticles and distributed them more uniformly to provide more active sites. Therefore, a superior hydrogen production rate of 11086.4 mL_H2_∙min^−1^∙g_Co_^−1^ and a low activation energy of 31.52 kJ mol^−1^ can be obtained for Co@150PC. Furthermore, it still maintained about 72.0% of the initial hydrogen production rate after 15 cycles. The DFT calculation results also indicated that Co@xPC can activate [BH_4_]^−^ molecules to promote the rapid dissociation of NaBH_4_ to release hydrogen. We also found the main reasons for the decrease in the catalytic performance of Co@150PC was the accumulation of borate as a by-product of the reaction during the test, which led to the formation of a strong adsorption (poly)borate shell on the surface of the Co nanoparticles. At the same time, the mutual fusion between the strongly adsorbed (poly)borate shells also weakened the dispersibility of Co nanoparticles, which further decreased the catalytic performance of Co@150PC.

## Figures and Tables

**Table 1:** The nitrogen adsorption–desorption measurement parameters of pure PC, Co and Co@150PC.

Catalyst	Specific Surface Area (m^2^·g^−1^)	Pore Volume (cm^3^·g^−1^)	Average Pore Diameter (nm)
PC	1527.499	0.530	3.837
Co	87.098	0.528	0.478
Co@150PC	274.101	0.348	1.429

**Table 2:** Comparison of the performance of different catalysts in catalyzing the NaBH_4_ hydrolysis reaction.

Catalyst Sample	Maximum Hydrogen Production Rate (mL_H2_∙min^−1^∙g_M_^−1^)	*E_a_* (kJ mol^−1^)	Durability	References
Co-Fe3O4@C	1403	49.2	59.3% after 5 cycles	[42]
CNSs@Pt0.1Co0.9	8943	38.0	85.12% after 5 cycles	[46]
Co-B/C	8033.89	56.72	-	[47]
Co–B/C	3887.1	56.37	25% after 6 cycles	[48]
Co–Mo–B/CC	1280.8	51.0	75.1% after 3 cycles	[49]
Ru–Co/C	9360	36.83	70% after 8 cycles	[50]
Co–B/MWCNT	5100	40.40	-	[51]
Co-B/N-C-700	2649	37.57	-	[52]
Co/PGO	5955	55.2	73% after 5 cycles	[53]
Co@NMGC	3575	35.2	82.5% after 20 cycles	[31]
Modified CCS/Co	11,600	33.4	-	[54]
CAs/Co	11,220	38.4	96.4% after 5 cycles	[55]
Co/C	530	44.1	-	[56]
Co@150PC	11,086.4	31.25	72% after 15 cycles	This work

## Data Availability

No data.

## Supplementary Materials

The following are available online at https://www.mdpi.com/article/10.3390/nano11123259/s1, Figure S1. TEM image of Co@150PC; Figure S2. XRD patterns of Co@50PC, Co@100PC and Co@200PC; Figure S3. Corresponding EDS mapping Co@150PC (a–d); Figure S4. C 1s XPS spectrum of Co@150PC; Figure S5. XRD patterns of Co@150PC and Co@150PC-15th.
