# Supplementary material for: Catalytic Hydrogen Evolution of NaBH4 Hydrolysis by Cobalt Nanoparticles Supported on Bagasse-Derived Porous Carbon
Source: Nanomaterials (Basel). 2021 Nov 30;11(12):3259. doi: 10.3390/nano11123259 (PMC8708045; doi:10.3390/nano11123259)
Supplement: Supplementary file 1 [file nanomaterials-11-03259-s001.zip › nanomaterials-1481763-supplementary.pdf]

Supplementary Material

Catalytic Hydrogen Evolution of $NaBH_4$ Hydrolysis by Cobalt Nanoparticles Supported on Bagasse-Derived Porous Carbon

Yiting Bu [1,2,†], Jiaxi Liu [1,†], Hailiang Chu [1], Sheng Wei [1,2], Qingqing Yin [1], Li Kang [1], Xiaoshuang Luo [1], Lixian Sun [1,2,*], Fen Xu [1,*], Pengru Huang [1,3], Federico Rosei [4], Aleskey A. Pimerzin [5], Hans Juergen Seifert [6], Yong Du [7] and Jianchuan Wang [7]

[1] Guangxi Key Laboratory of Information Materials and Guangxi Collaborative Innovation Center of Structure and Property for New Energy and Materials, School of Material Science & Engineering, Guilin University of Electronic Technology, Guilin 541004, China; ytb11727012550@163.com (Y.B.); jxliu2019@126.com (J.L.); chuhailiang@guet.edu.cn (H.C.); ws1801101003@163.com (S.W.); yqq15870030656@163.com (Q.Y.); kangli000hello@163.com (L.K.); shirley_lxs@126.com (X.L.); pengruhuang@guet.edu.cn (P.H.)

[2] School of Mechanical & Electrical Engineering, Guilin University of Electronic Technology, Guilin 541004, China

[3] Department of Materials Science and Engineering, National University of Singapore, Singapore 117575, Singapore

[4] Centre for Energy, Materials and Telecommunications, Institut National de la Recherche Scientifique, 1650 Boulevard Lionel-Boulet Varennes, Québec, QC J3X 1S2, Canada; rosei@emt.inrs.ca

[5] Chemical Department, Samara State Technical University, 443100 Samara, Russia; al.pimerzin@gmail.com

[6] Karlsruhe Institute of Technology, Institute for Applied Materials-Applied Materials Physics, Hermann-von-Helmholtz-Platz 1, 76344 Eggenstein-Leopoldshafen, Germany; hans.Seifert@kit.edu

[7] State Key Laboratory of Powder Metallurgy, Central South University, Changsha 410083, China; yong-du@csu.edu.cn (Y.D.); jcw728@126.com (J.W.)

* Correspondence: sunlx@guet.edu.cn (L.S.); xufen@guet.edu.cn (F.X.)

† Yiting Bu and Jiaxi Liu contributed equally.

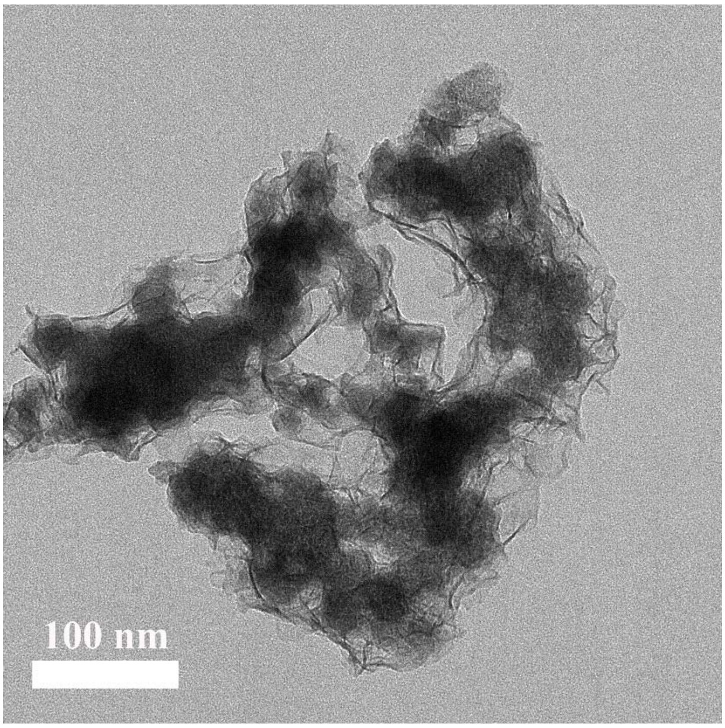

Figure S1. TEM image of Co@150PC.

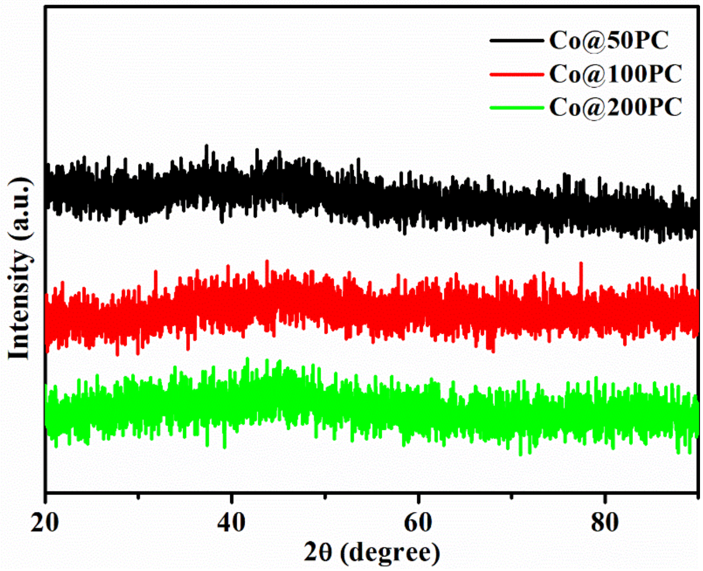

Figure S2. XRD patterns of Co@50PC, Co@100PC and Co@200PC.

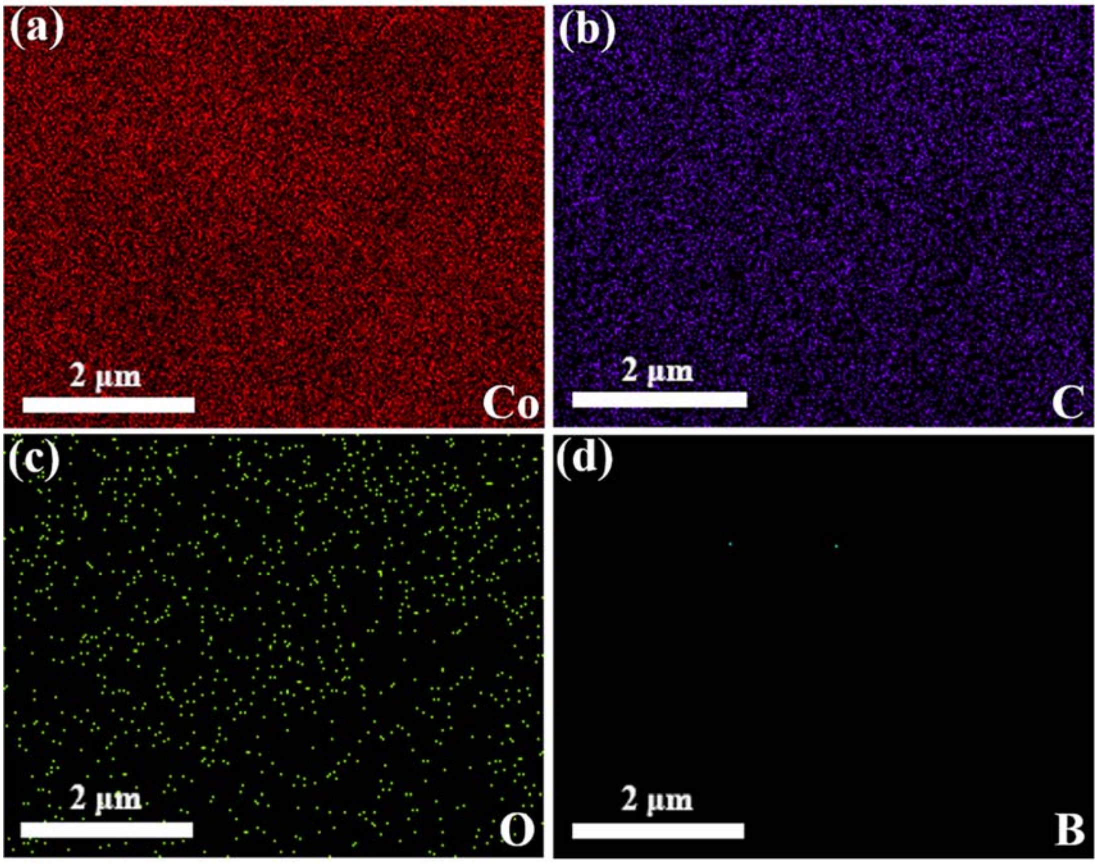

Figure S3. Corresponding EDS mapping Co@150PC (**a**–**d**).

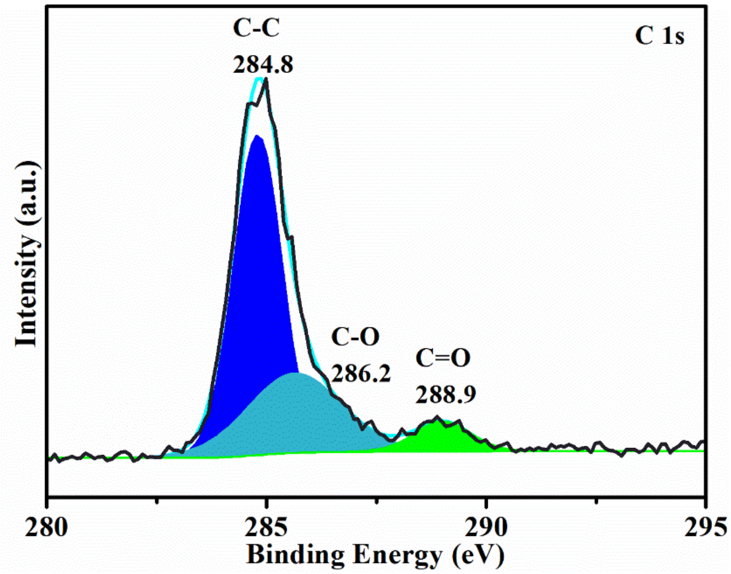

Figure S4. C 1s XPS spectrum of Co@150PC.

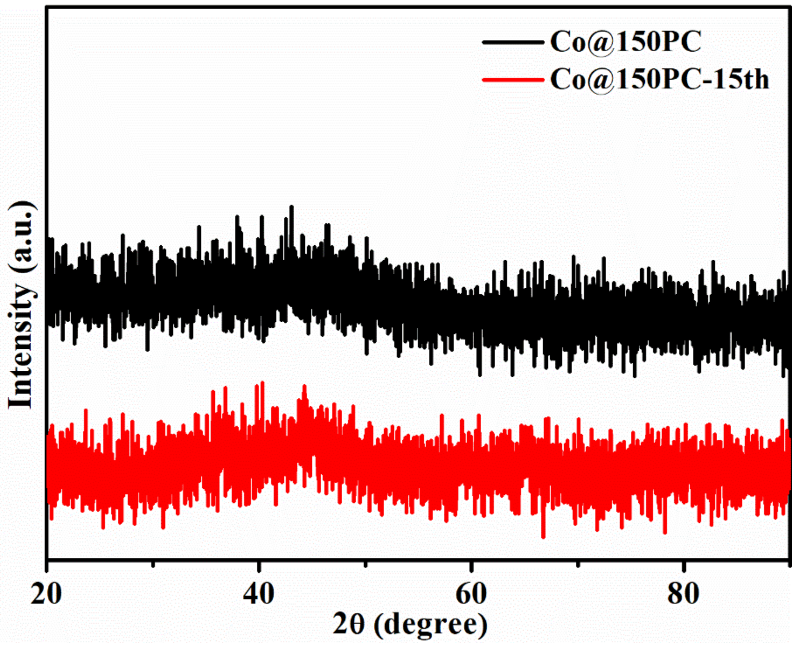

Figure S5. XRD patterns of Co@150PC and Co@150PC-15th b.
